# Polyphenolic Antioxidants from Agri-Food Waste Biomass

**DOI:** 10.3390/antiox8120624

**Published:** 2019-12-06

**Authors:** Dimitris P. Makris, Selin Şahin

**Affiliations:** 1 Green Processes & Biorefinery Group, School of Agricultural Sciences, University of Thessaly, 43100 Karditsa, Greece; 2 Chemical Engineering Department, Istanbul University-Cerrahpaşa, 34320 Avcilar, Istanbul, Turkey; selins@istanbul.edu.tr

As the world’s population is rapidly expanding, environmental aggravation and bioresource depletion are becoming challenges of paramount importance. Agricultural production and the food supply chain are major sources of waste biomass, which poses an unprecedented risk to land and water pollution, and eventually, public health. However, agri-food processing residues, owed to their peculiar composition, are also recognized as materials of high biorefinery potency, offering a range of opportunities for sustainable production of food, feed, chemicals, and energy.

In such a framework, the valorization of by-products and wastes originating from the agricultural and food sector for the production of a broad spectrum of high value-added products has emerged as a key strategy. The exploitation of agri-food side streams by implementing eco-friendly and cost-effective technologies is now considered as a primary route towards zero-waste production, and the design and commercialization of novel bio-based formulations has proven to be significantly more profitable than biofuel production [1].

Polyphenols are bioactive secondary plant metabolites possessing versatile properties, including long-term protection from cardiovascular diseases, chemoprotective activity, antioxidant and anti-inflammatory potency. The recovery of polyphenolic substances from agri-food wastes is a primary target in the higher-value options of biorefining, and numerous investigations have been and are being carried out towards this direction [2]. The objective of this special issue is a compilation of the most recent, state-of-the-art studies pertaining to the valorization of food processing wastes to highlight cutting-edge advances in the field.

Kim et al. [3] investigate the phenanthrene content of waste peels from a popular Asian food, the Chinese yam (*Dioscorea batatas*). Phenanthrenes are major polyphenolic substances in yam, and their content may define its nutrition value. The authors, after performing a detailed High-performance liquid chromatography (HPLC) analysis, conclude that yam peels may be a source rich in phenanthrenes, and thus they may merit greater attention as a bioresource of functional phytochemicals.

Alañón et al. [4] study the extracts from mango by-products (peel, husk seed, and seed) for potential bioactivity. The results drawn showed that mango seed extract afforded a 72% percentage inhibition of platelet aggregation induced by adenosine 5’-diphosphate (ADP) agonist in a dose-dependent manner. The most potent extract contained monogalloyl compounds, tetra- and penta-galloylglucose, ellagic acid, mangiferin, and benzophenones such as maclurin derivatives and iriflophenone glucoside. Out of these compounds, mangiferin exhibited an inhibitory effect of 31%, suggesting its key role as one of the main contributors to the antiplatelet activity of mango seed. The authors suggest that mango seed could be a bioresource of compounds with antiplatelet properties and may be used for designing functional foods.

Di Mauro et al. [5] examine the possibility of using olive mill wastewater (OMW) to produce ophthalmic nutraceutical formulations. Various adsorbents were tested to selectively recover a polyphenol-rich fraction, which was then assayed for cytotoxicity and antioxidant/anti-inflammatory activities through in vitro tests. The results indicate that the fraction (0.01%) had no toxic effects and was able to protect cells against oxidant and inflammatory stimulus, reducing reactive oxygen species and TNF-α levels. The authors also prepared a novel stable ophthalmic hydrogel containing a polyphenolic fraction (0.01%) and assessed the technical and economic feasibility of the process at a pre-industrial level.

Birsan et al. [6] study the effective recovery of antioxidant polyphenols from light, dark, and mix brewer’s spent grain (BSG) using conventional maceration, microwave, and ultrasound-assisted extraction. Irrespective of the extraction methods used, the saponification of BSG yielded higher polyphenols than in the crude extracts, with the EtOAc fractionations being the most effective. Microwave and ultrasound-assisted extractions did not improve the total polyphenol yield when compared to the conventional maceration method. The authors conclude that BSG light may be regarded as a sustainable, low-cost source of natural antioxidants, tapped for applications in the food and phytopharmaceutical industries.

Matos et al. [7] investigate winemaking waste streams as a material with cosmeceutical potential. The authors produced extracts from grape marc and wine lees, using solid–liquid (SL) extraction with and without microwave (MW) pretreatment, and assayed them for antioxidant activity through chemical (ORAC/HOSC/HORAC) and cell-based (keratinocytes—HaCaT; fibroblasts—HFF) tests. Their inhibitory capacity towards specific enzymes involved in skin ageing (elastase; MMP-1; tyrosinase) was also appraised. MW pretreatment prior to conventional SL extraction led to overall better outcomes. Red wine lees extracts presented the highest phenolic content and exhibited the highest antioxidant capacity, being also the most effective inhibitors of elastase, MMP-1, and tyrosinase. The authors argue that winemaking waste streams could be valuable sources of natural ingredients for cosmeceutical applications.

Martín-García et al. [8] focus their research on the establishment of ultrasound-assisted extraction of proanthocyanidin compounds from brewing spent grains using a sonotrode. Response surface methodology was used to study the effects of three factors, namely, solvent composition, time of extraction, and ultrasound power. The highest content of proanthocyanidins was obtained using 80/20 acetone/water (*v/v*), 55 min, and 400 W. The authors support that this methodology allows for the extraction of 1.01 mg/g dry weight of pronthocyanidins from brewer’s spent grain, this value being more than two times higher than conventional extraction.

Abi-Khattar et al. [9] present an innovative technology for effective polyphenol recovery from olive leaves, namely Ired-Irrad^®^. In this study, optimization of infrared-assisted extraction was conducted using response surface methodology to intensify polyphenol recovery from olive leaves. The extraction efficiency using Ired-Irrad^®^, a newly patented infrared apparatus (IR), was compared to the water bath (WB) conventional extraction. Under optimal conditions, the total phenolic content yield was enhanced by more than 30% using IR as contrasted to WB, which required 27% more ethanol consumption. The extraction of two major phenolic compounds of the leaves, oleuropein and hydroxytyrosol, was intensified by 18% and 21%, respectively. IR extracts increased the antiradical activity by 25% and the antioxidant capacity by 51% compared to WB extracts. On the other hand, extracts of olive leaves obtained by both techniques exhibited equal effects regarding the inhibition of 20 strains of *Staphylococcus aureus*. Similarly, both extracts inhibited aflatoxin B1 (AFB1) secretion by *Aspergillus flavus*, with no growth inhibition of the fungus. The authors claim that this innovative technique allows for significantly reduced energy and solvent consumption while maintaining a similar quantity and quality of phenolic compounds as what is optimally obtained using WB.

Fernandez et al. [10] propose apple pomace as a sustainable food ingredient. The authors used acidified hot water extraction as a clean, feasible, and easy approach for the recovery of polyphenols. This technique allowed them to obtain 296 g of extract per kg of dry apple pomace, including 3.3 g of polyphenols and 281 g of carbohydrates. Ultrafiltration and solid-phase extraction using C18 cartridges of the hot water extract suggested that, in addition to the apple native polyphenols, polyphenols could also be present as complexes with carbohydrates. For the water-soluble polyphenols, antioxidant and anti-inflammatory effects were observed by inhibiting chemically generated hydroxyl radicals (OH•) and nitrogen monoxide radicals (NO•) produced in lipopolysaccharide-stimulated macrophages. The water-soluble polyphenols, when incorporated into yogurt formulations, were not affected by fermentation and improved the antioxidant properties of the final product.

Li et al. [11] propose the use of edible oils as effective means of recovering volatile and non-volatile compounds from rosemary since these materials can serve as food-grade solvents. Soybean oil could obtain the highest total phenolic compounds among 12 refined oils including grapeseed, rapeseed, peanut, sunflower, olive, avocado, almond, apricot, corn, wheat germ, and hazelnut oils. The addition of oil derivatives to soybean oils, such as glyceryl monooleate, glyceryl monostearate, diglycerides, and soy lecithin, not only significantly enhanced the oleo-extraction of non-volatile antioxidants by 66.7% approximately but also help to remarkably improve the solvation of volatile aroma compounds by 16% in refined soybean oils. These results were in good consistency with their relative solubilities predicted by the more sophisticated COSMO-RS (COnductor like Screening MOdel for Real Solvents) simulation. According to the authors, the use of vegetable oils and their derivatives as bio-based solvents for the extraction yield of natural antioxidants and flavors from rosemary show an important potential in up-scaling. Along with the integration of green techniques (e.g., ultrasound, microwave), such technologies can contribute towards zero-waste biorefinery from biomass waste and the production of high value-added extracts in future functional food and cosmetic applications.

Finally, Lakka et al. [12] deliver an investigation on saffron processing wastes as a bioresource of high value-added compounds and the development of a green extraction process for polyphenol recovery using a natural deep eutectic solvent. The study included an appraisal of the molar ratio of hydrogen bond donor/hydrogen bond acceptor in order to come up with the most efficient DES composed of l-lactic acid/glycine (5:1), and then optimization of the extraction process using response surface methodology. Under optimal conditions, the extraction yield in total polyphenols achieved was 132.43 ± 10.63 mg gallic acid equivalents per g of dry mass. The temperature assay suggested that extracts displayed maximum yield and antioxidant activity at 50–60 °C. Liquid chromatography-mass spectrometry analysis of the SPW extract obtained under optimal conditions showed that the predominant flavonol was kaempferol 3-*O*-sophoroside and the major anthocyanin delphinidin 3,5-di-*O*-glucoside. The results indicate that SPW extraction with the DES used is a green and efficient methodology and may afford extracts rich flavonols and anthocyanins, which are considered to be powerful antioxidants.
